# Supplementary material for: Systematic computational assessment of atrial function impairment due to fibrotic remodeling in electromechanical properties
Source: PLoS Comput Biol. 2025 Dec 5;21(12):e1013265. doi: 10.1371/journal.pcbi.1013265 (PMC12680338; doi:10.1371/journal.pcbi.1013265)
Supplement: S1 Text — Supplementary text and figures providing further details of methods, underlying data, and extended results. (PDF) [file pcbi.1013265.s001.pdf]

S1 Text: Appendix

A Calibration of electrical stimulus locations and personalized CV values

We obtained LA EAM during the procedure for each patient, from which we used sinus rhythm maps for calibration of personalized CV values. A CARTO system (J&J MedTech) was used to acquire EAM data during the procedure, and the open-source software OpenEP [1] subsequently used to extract local activation time maps.

We used the EAM data to identify early activation sites by delineating tissue regions activated within the first 5 ms. For all three patients, this resulted in three disjoint regions. Data for Patient 3 displayed earliest activation for the left superior pulmonary vein; however there was no propagation out of this area and therefore filtered out by thresholding (gray areas in Fig C, bordered by red and yellow). Next, we manually identified the corresponding areas on the volumetric geometries derived from the LGE surfaces. These areas were set as electrical stimulus (pacing) locations in all organ-scale simulations performed.

Next, we estimated pacient-specific CV values by changing model CV values such that the total LA activation time predicted by the EP model simulation matched the value extracted from the CARTO data. We used a constant anisotropy ratio of $1 : 1/\sqrt{5}$ for $\mathrm{CV_L}$:$\mathrm{CV_T}$ values, following Zahid et al. [2, 3], such that we only had one varying parameter ($\mathrm{CV_L}$). We then ran EP simulations, measured the total LA activation time, and updated the CV values until the total activation time matched the EAM-recorded total activation time within 1 ms.

The algorithm (the iterative process) used to determine optimal CV values for each patient is written out below. In this, the input parameters were a patient-specific geometry, electrical stimulus locations, and total activation time $totalacttime_{EAM}$ derived from the EAM data. Optimized CV values were read out as output. Each EP simulation was performed across corresponding patient-specific geometry over a single cardiac beat. The increase and decrease of CV_L were determined manually – while this could also be determined by appropriate incremental increases, we found it easier to do gradual changes manually since we only had three cases. We did not change any parameters in the fibrotic regions of the patient geometry, assuming that the difference between fibrotic and non-fibrotic configurations would be minor for total activation times.

Fig A–C display spatial plots used to determine earliest activation (pacing) locations from three different angles. For all three figures, in (A) we display the activation time derived from the EAM (CARTO) data. In (B) we show the same data, but with colors adjusted to highlight the first 5 ms of activation only. In (C) we show the locations we picked on the volumetric meshes. The electrical stimulus locations were determined manually by matching the area determined by the 5 ms activation area as closely as possible, based on landmarks and geometrical features observed across both geometries. In (D) we display the activation times simulated with our EP model, using electrical stimulus locations from (C) and with optimal CV values as listed in Table 1 in the main text (i.e., healthy baseline values) and in Table A.

Algorithm 1: Patient-specific CV calibration

Input : patient geometry, electrical stimulus locations, $totalacttime_{EAM}$

$CV_L \leftarrow 1.2$ /* initial guess */ ;

do

  $CV_T \leftarrow CV_L / \sqrt{5}$;

  $totalacttime_{SIM} \leftarrow$ EP simulation(patient geometry, electrical stimulus locations, CV_L, CV_T);

  if $totalacttime_{CARTO} < totalacttime_{SIM}$ **then**

    Increase CV_L ;

  else if $totalacttime_{SIM} < totalacttime_{EAM}$ **then**

    Decrease CV_L ;

  end

while $abs(totalacttime_{CARTO} - totalacttime_{SIM}) < 1$;

B Patient-specific CV values subject to fibrotic changes

In Table A, we list CV values imposed from all combinations of reduced and baseline values of all EP parameters considered in our analysis. Baseline CV values were calibrated on a patient-specific basis, as previously described. Fibrotic values for longitudinal and transverse reductions were calculated by dividing the values by 0.4461 and 0.3526, respectively, as described in the main text. Next, we used open-source software *tuneCV* functionality [4] from the *openCARP* project [5] to estimate the corresponding conductance values for all four values (CV_L and CV_T; non-fibrotic and fibrotic). The conductance values, combined with altered ion channel conduction values, were then used to calculate the corresponding changes in prescribed CV values.

Table A. CV values for all combinations of baseline/reduced conductance and baseline/reduced longitudinal/transverse CV for Patient 1 (P_1), Patient 2 (P_2), and Patient 3 (P_3).

		Baseline CV (m/s)			Reduced CV (m/s)		
		P_1	P_2	P_3	P_1	P_2	P_3
Conduction values	CV_L	1.190	1.900	0.835	0.782	1.249	0.549
at baseline	CV_T	0.532	0.848	0.373	0.277	0.442	0.194
$I_{Na}\times0.6$	CV_L	0.947	1.532	0.653	0.610	0.995	0.418
	CV_T	0.403	0.664	0.276	0.199	0.331	0.157
$I_{CaL}\times0.5$	CV_L	1.186	1.897	0.828	0.775	1.245	0.539
	CV_T	0.520	0.841	0.359	0.261	0.429	0.177
$I_{K1}\times0.5$	CV_L	1.221	1.930	0.868	0.815	1.280	0.583
	CV_T	0.565	0.880	0.408	0.312	0.476	0.230
$I_{Na}\times0.6$, $I_{CaL}\times0.5$	CV_L	0.942	1.529	0.645	0.601	0.990	0.405
	CV_T	0.390	0.656	0.258	0.178	0.316	0.132
$I_{Na}\times0.6$, $I_{K1}\times0.5$	CV_L	0.980	1.563	0.689	0.646	1.028	0.456
	CV_T	0.442	0.700	0.316	0.240	0.370	0.176
$I_{CaL}\times0.5$, $I_{K1}\times0.5$	CV_L	1.217	1.927	0.862	0.809	1.276	0.575
	CV_T	0.557	0.874	0.397	0.300	0.466	0.218
$I_{Na}\times0.6$, $I_{CaL}\times0.5$, $I_{K1}\times0.5$	CV_L	0.975	1.560	0.682	0.638	1.023	0.446
	CV_T	0.431	0.692	0.303	0.227	0.359	0.162

C Average reported metrics for all FFD combinations

In Fig D and E, we include average results for all runs in the FFD in a combined heatmap/tabular format. In this, we first normalized all reported metrics first relative to patient baseline values, then averaged across the three patients.

Combinations 9–16 and 25–32 imposed an impairment of I_{CaL}. In reported metrics, this was reflected by an overall decrease in A-loop area, booster function, reservoir function, and upstroke pressure difference. Combinations 4–8 and 21–24 involved an impairment of I_{K1} but not of I_{CaL}, in which there was an overall increase in metric values. Combinations 13–16 and 29–32 involved an impairment of both, and there was a slight mitigating effect among these compared to Combinations 9–13 and 25–28.

D Main effect for elevated fibrosis

Main effect plots for all FFD combinations are included in Fig F, showcasing original versus 50% elevated fibrosis. For both original and elevated fibrosis, we compared all combinations in which the given parameter was set to baseline (B) levels versus those set to fibrotic (F) levels. Plots corresponding to factor found to be statistically significant are also included in the main paper (Fig. 13).

Comparing original to elevated fibrosis levels, a greater drop was observed across most parameters and metrics (A-loop area, booster function, reservoir function, and upstroke pressure difference), while conduit function did not change much. For impaired I_{ICaL} the elevated fibrotic comparison was higher (the slope steeper) than in the original fibrosis comparison.

References

1. Williams SE, Roney CH, Connolly A, Sim I, Whitaker J, O'Hare D, et al. OpenEP: a cross-platform electroanatomic mapping data format and analysis platform for electrophysiology research. Frontiers in Physiology. 2021;12:646023.

2. Zahid S, Cochet H, Boyle PM, Schwarz EL, Whyte KN, Vigmond EJ, et al. Patient-derived models link re-entrant driver localization in atrial fibrillation to fibrosis spatial pattern. Cardiovascular Research. 2016;110(3):443–454.

3. Bifulco SF, Scott GD, Sarairah S, Birjandian Z, Roney CH, Niederer SA, et al. Computational modeling identifies embolic stroke of undetermined source patients with potential arrhythmic substrate. Elife. 2021;10:e64213.

4. Costa CM, Hoetzl E, Rocha BM, Prassl AJ, Plank G. Automatic parameterization strategy for cardiac electrophysiology simulations. In: Computing in Cardiology 2013. IEEE; 2013. p. 373–376.

5. Plank G, Loewe A, Neic A, Augustin C, Huang YL, Gsell MA, et al. The openCARP simulation environment for cardiac electrophysiology. Computer methods and Programs in Biomedicine. 2021;208:106223.

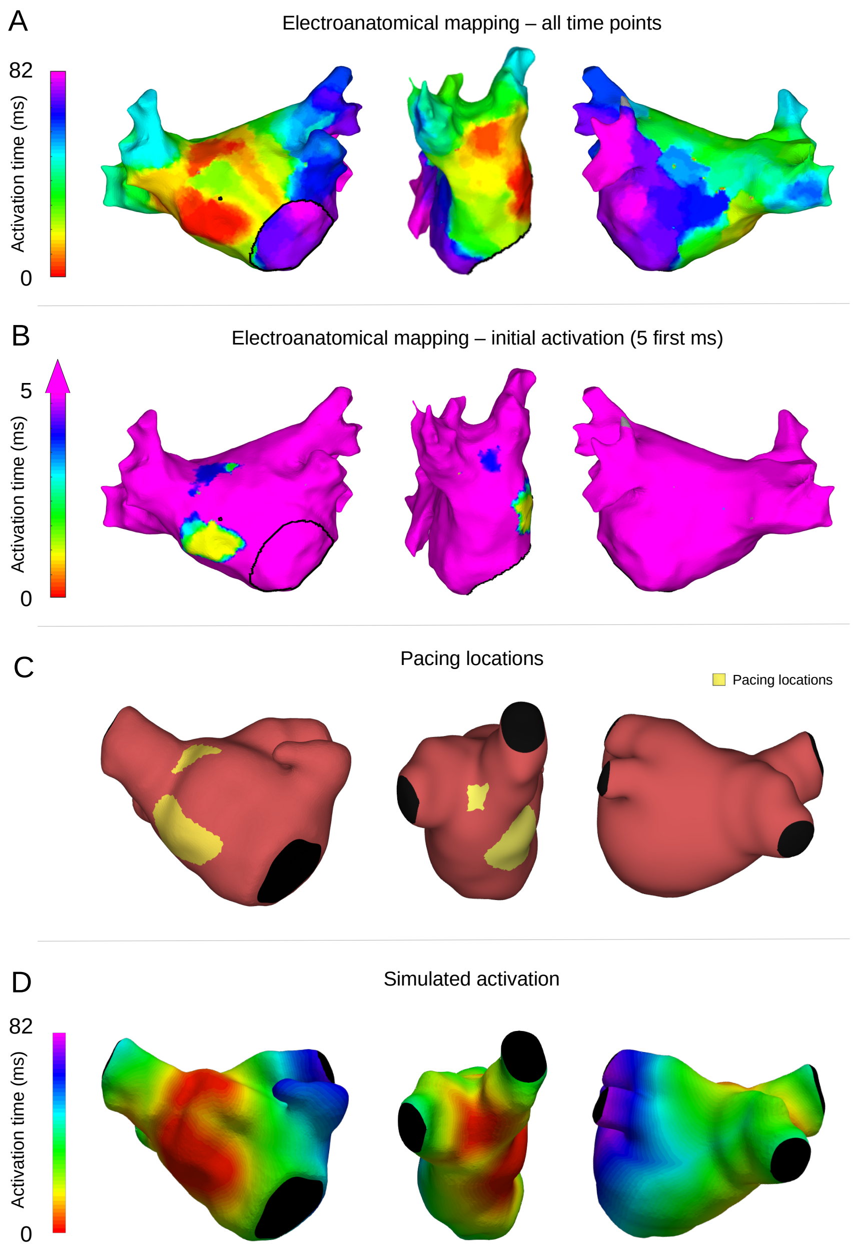

Fig A. Patient 1 activation times and electrical stimulus locations. (A) Activation times derived from the EAM (CARTO) data, (B) activation times for the first 5 ms, (C) pacing locations, and (D) simulated activation times for Patient 1.

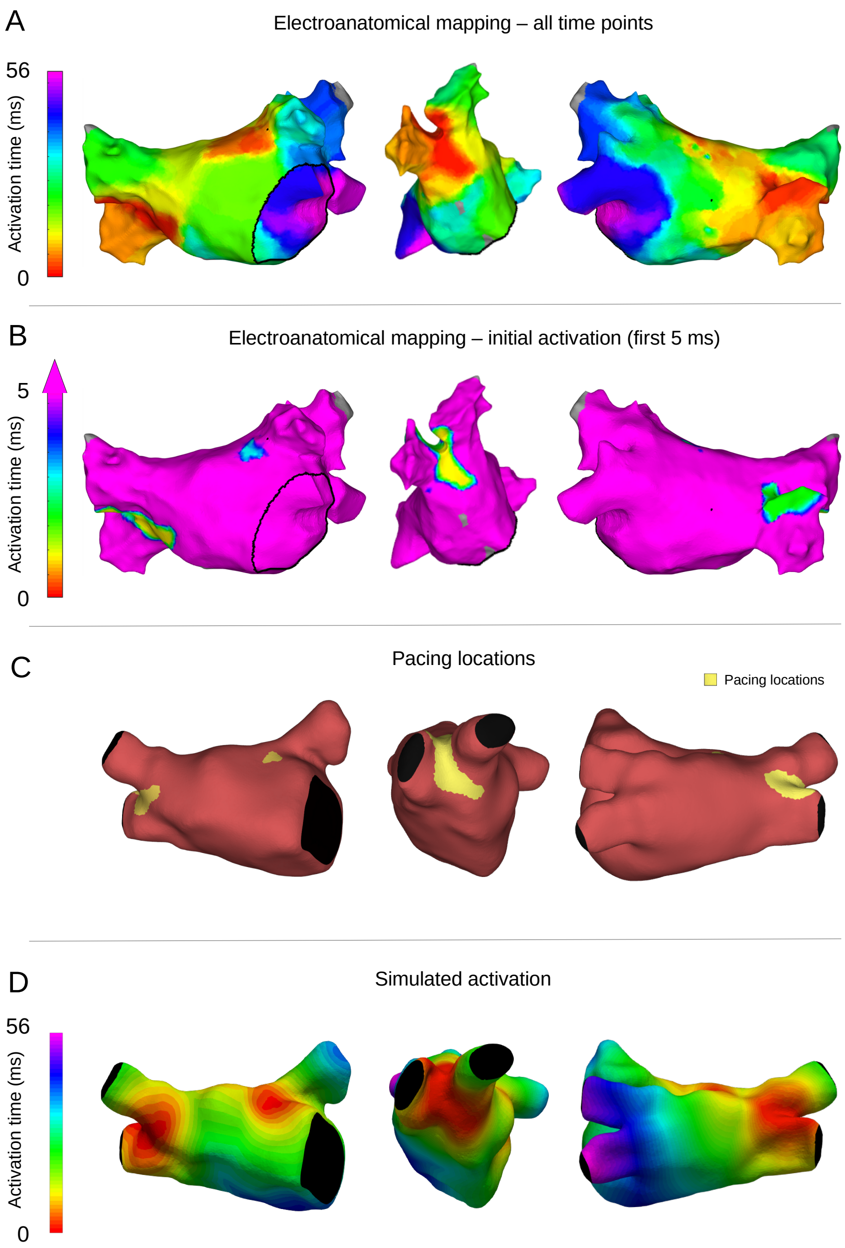

Fig B. Patient 2 activation times and electrical stimulus locations. (A) Activation times derived from the EAM (CARTO) data, (B) activation times for the first 5 ms, (C) pacing locations, and (D) simulated activation times for Patient 2.

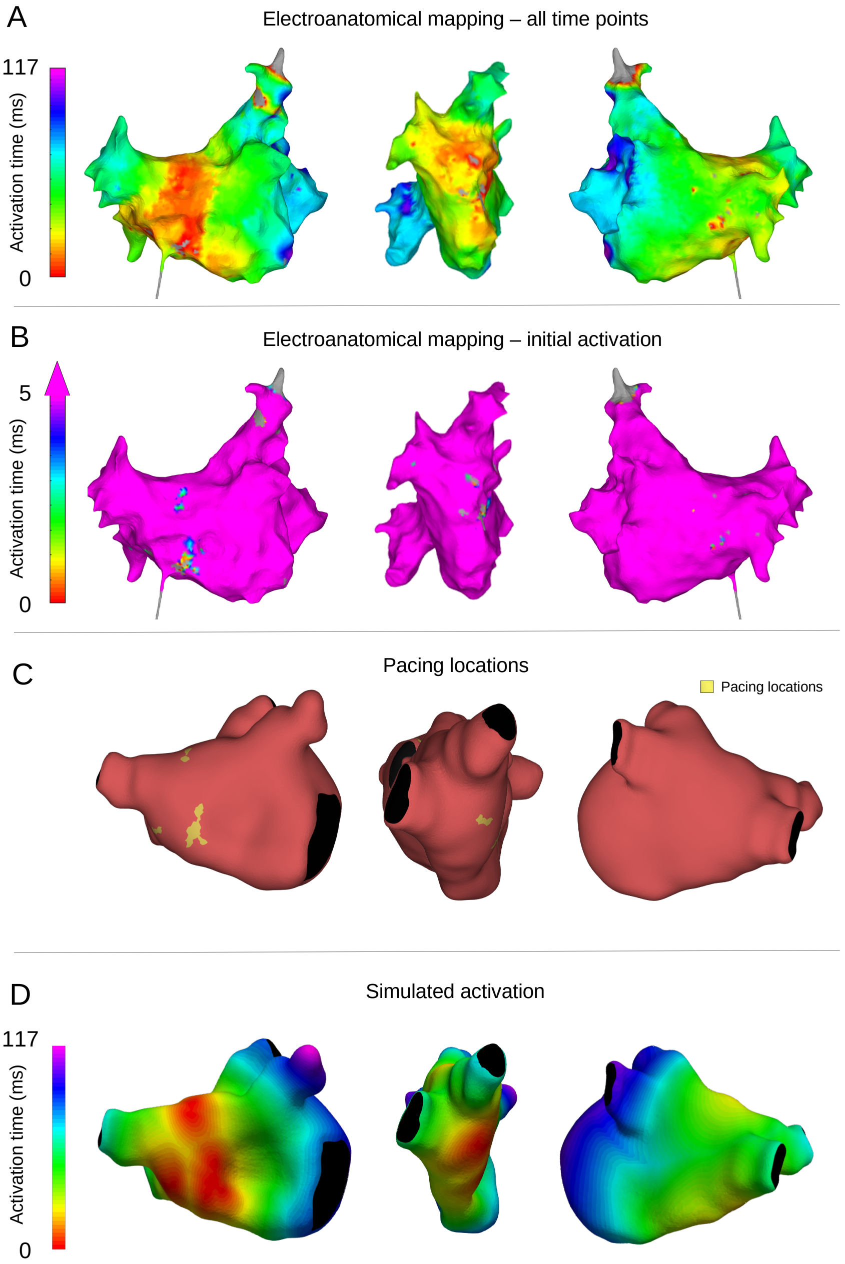

Fig C. Patient 3 activation times and electrical stimulus locations. (A) Activation times derived from the EAM (CARTO) data, (B) activation times for the first 5 ms, (C) pacing locations, and (D) simulated activation times for Patient 3.

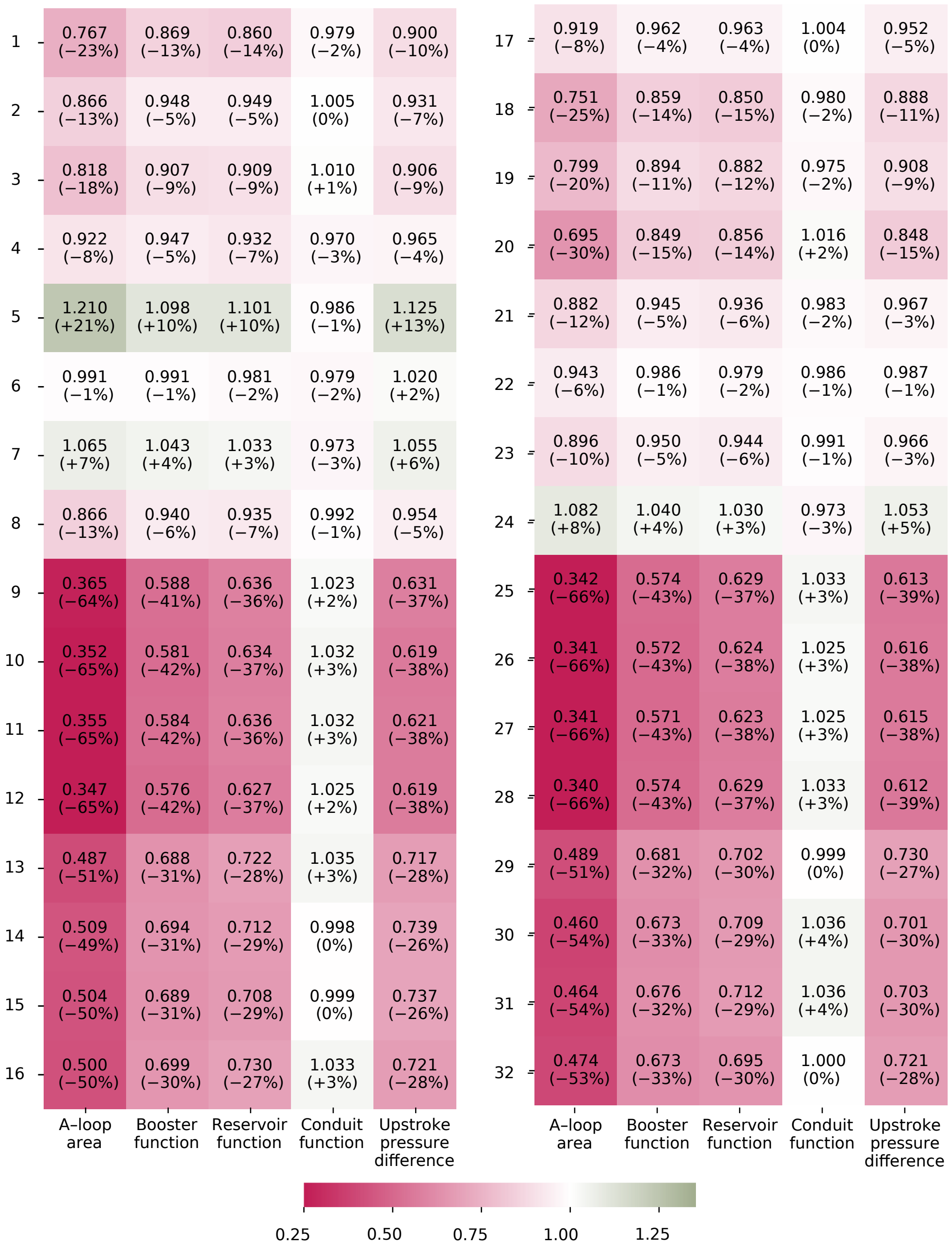

Fig D. Metrics for FFD Combinations 1–32; original fibrosis burden. Average (across all three patients) normalized (relative to baseline values) metrics for each parameter combination. Corresponding percentage change is listed in parenthesis.

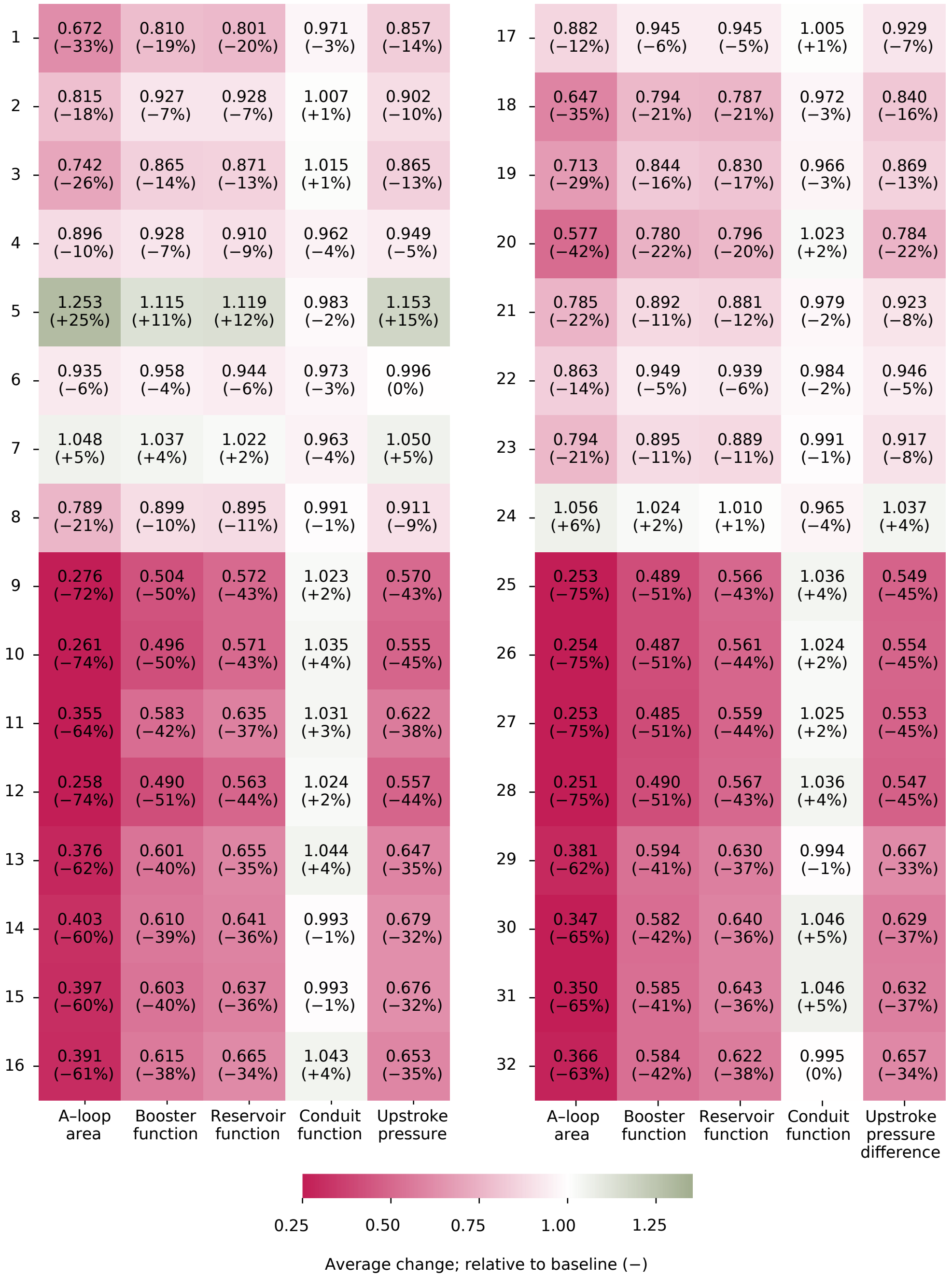

Fig E. Metrics for FFD Combinations 1–32; 50% synthetically elevated fibrosis burden. Average (across all three patients) normalized (relative to baseline values) metrics for each parameter combination. Corresponding percentage change is listed in parenthesis.

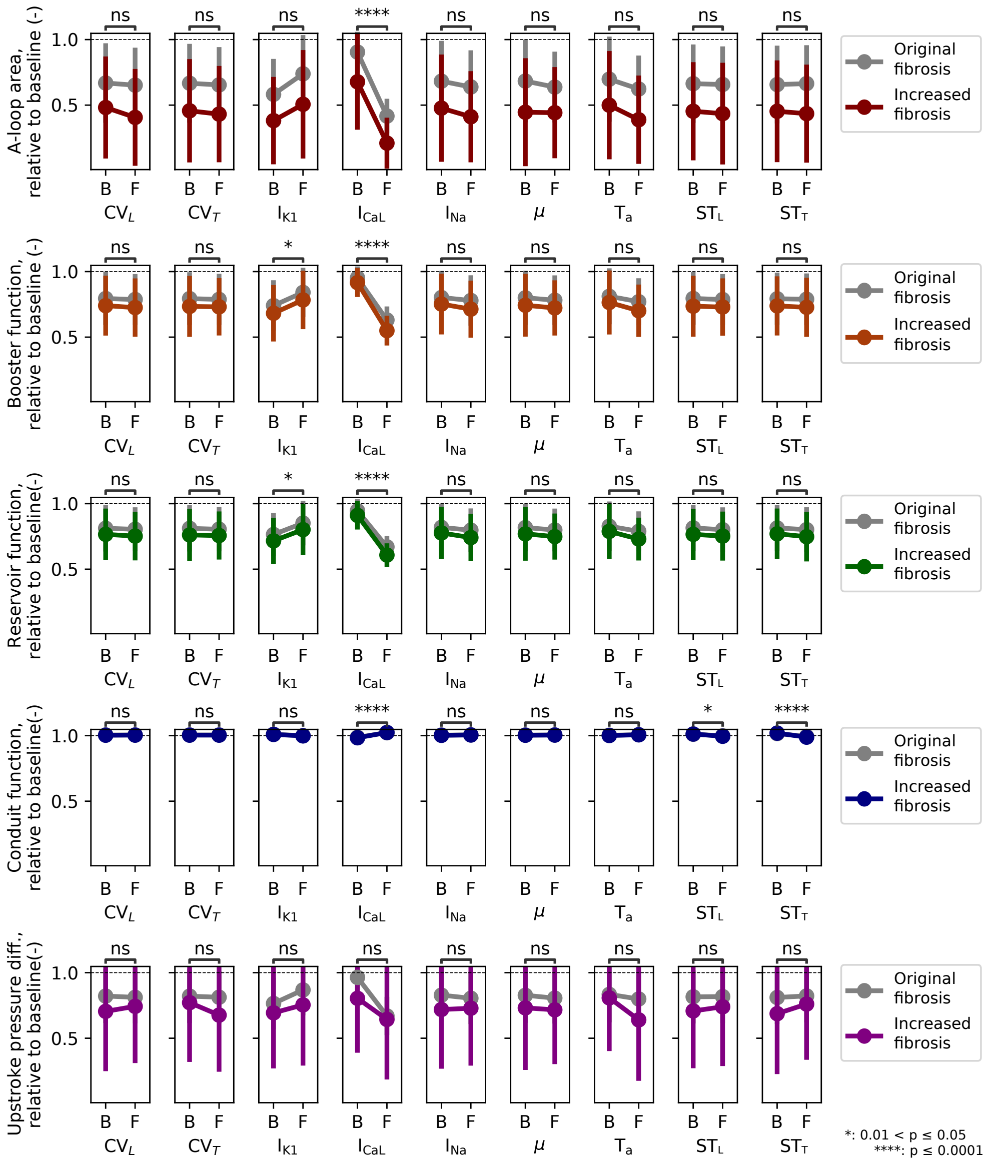

Fig F. FFD main effect plots, original and 50% synthetically elevated fibrosis. Error bars indicate standard deviation, and gray plots represent original fibrosis levels (based on the same underlying data as in Fig 13 in the main text). Comparisons for significant differences were performed for elevated fibrosis simulations.
