# Supplementary material for: Systematic computational assessment of atrial function impairment due to fibrotic remodeling in electromechanical properties
Source: PLoS Comput Biol. 2025 Dec 5;21(12):e1013265. doi: 10.1371/journal.pcbi.1013265 (PMC12680338; doi:10.1371/journal.pcbi.1013265)
Supplement: Attachment — Submitted filename: response_letter.pdf [file pcbi.1013265.s005.pdf]

Dear Editors,

We would like to thank the reviewers for the effort taken to review our manuscript and for their valuable comments and suggestions. We have carefully considered each point and made revisions accordingly. Our responses are provided below, with replies highlighted in violet and the corresponding manuscript changes in a blue. These changes are marked directly in the manuscript, also in blue (in which red text also displays removed text).

Reviewer 1

Telle et al. use an electromechanical model to quantify the relative contributions of fibrotic remodeling on left atrial function. They identified two calcium-related properties that explain most the reduction in atrial function following fibrosis. The use of the model to explain why these parameters are consequential for LA function is much appreciated. The study design is sound, manuscript is well-written, and figures support the methods and findings and are clearly labeled. Congratulations on the authors for this insightful work. However, I have several comments that need to be addressed, which hopefully will further strengthen the manuscript.

Major comments:

- The choice of patients could significantly influence the study outcomes, with substantial differences already observed between the baseline properties (Table 1) of the three selected patients. Was there a rationale for choosing these three patients? Were the patients selected from a larger cohort? Please provide a more detailed description of the patient recruitment.

  We appreciate the reviewer's point and agree that patient-specific characteristics may influence our results. The three patients considered were prospectively screened for catheter ablation at the University of Washington Medical Center and selected based on predefined criteria to ensure complete, co-registered datasets (geometry, fibrosis distribution, and electroanatomical mapping). While CARTO data was not strictly essential for the sensitivity analysis and additional geometries from earlier cohorts could have been included, the calibration and simulation processes are time-intensive. Furthermore, we have extensive baseline characteristics, which are not patient-specific but rather are based on average human physiology. This ensures that our results are at least somewhat general. While the specific results might change, the use of more patients would likely not greatly change the main takeaways of our study (i.e., prominent roles played by key electrophysiological derangements in fibrotic regions). We therefore limited this study to these three patients, but are aiming to expand the analysis to more patients in future work.

  We have expanded upon patient selection criteria as follows: (line 161–173)

  Patients were directly recruited for this and subsequent studies, and not part of earlier cohorts. We included AF patients undergoing ablation procedures, with availability of LGE-MRI suitable for LA wall segmentation and fibrosis quantification, and indicated high-density EAM of the LA performed within a 1-month time-frame of the MRI scan. Exclusion criteria were prior atrial ablation, prior cardiac surgery, contraindications to MRI or gadolinium contrast (e.g., device incompatibility, severe renal dysfunction, pregnancy, gadolinium sensitivity), and inability to obtain diagnostic-quality images due to arrhythmia or habitus constraints. The recruited patients included in this study all satisfied pre-specified data-quality requirements (diagnostic LGE-MRI enabling fibrosis segmentation and EAM with adequate LA surface coverage and point density).

  The low sample size is also commented on under limitations (line 696–699):

  We only consider three patients, whereas clinical studies typically involve hundreds of patients. Combined MRI and EAM data collection is logistically challenging, while model simulations are

time-consuming. Extending the analysis to a larger cohort would increase confidence in our findings and allow more granular analysis.

- LA EDV for was around two-fold greater for Patients 1 and 2 compared to Patient 3. Was this because of remodeling or body size? Reporting body surface area (or another related metric) in Table would be helpful for this.

  We thank the reviewer for raising this point. To address whether differences in volume reflect true remodeling versus body size, we now report body surface area (BSA) and LA EDV indexed to BSA (LAEDVi, mL/m^2) in Table 1. Indexing attenuated but did not eliminate inter-patient differences. Patients 1 and 2 had larger LAEDVi than Patient 3, supporting a contribution from LA remodeling beyond body size; however all three subjects had enlarged left atria relative to normal reference values.

  We also comment on these differences in the discussion (line 698–703):

  Two of our three patients (Patients 1 and 2, with LA volume indices 55.5 and 48.3) had enlarged LAs, with end-diastolic volumes and volume indices above normal ranges [1]. Using the Utah classification standard, Patients 1 and 3 had fibrosis burden at level II (5–20%), while Patient 2 was at level III (20–35%).

- Related to the previous comment: a constant wall thickness was assumed in all patients. However, would a different wall thickness be expected between Patients 1 and 2 and Patient 3?

  The reviewer again raises an interesting point. We went back to Cine MR images and extracted ranges of minimum and maximum wall thickness for each patient, which are now reported in Table 1 (with min–max ranges being 1.4–2.4 mm, 2.3–3.0 mm, and 1.6–2.4 mm for Patient 1–3, respectively). We could potentially have used an average of these as patient-specific thickness; however, it is unlikely that the modification would have any noticeable effect. We have commented on this in the Methods as follows (line 182–184):

  We used a uniform thickness following literature rather than personalized values (with ranges reported in Table 1), as we did not have spatial thickness values co-registered with the geometries.

  More accurate representations can be obtained, as in other LA studies. We have commented on this aspect in the discussion in the following paragraph, highlighting patient-specific and regionally varying wall thickness as potential future avenues (line 757–765):

  The geometrical assumptions introduce additional limitations. We used a uniform myocardial thickness of 2 mm, although atrial wall thickness varies regionally and between patients [2], see also reported ranges in Table 1. Changes in LA volume, fibrosis burden, and wall thickness are all components of LA remodeling. Relationships between these components remain active research areas, with implications for atrial pump function and AF substrate development [3, 4, 2, 5]. In addition, myofiber architecture was specified using a rule-based algorithm. Cardiac computed tomography offers the ability to quantify regional wall thickness and patient-specific myofiber orientation [6]. Incorporating such data could enhance model accuracy and predictive value.

- It is not completely clear to me how the 50% fibrotic burden was achieved. Was the threshold lowered until the target total burden was achieved?

  We apologize for the confusion. The reviewer is effectively correct. In our workflow, the target total fibrosis burden is achieved by identifying an optimal cutoff for normalized LGE values using the priority-based method we describe in Methods. To achieve the models with artificially increased fibrosis (+50%), we modified the target total burden and identified the new corresponding optimal cutoff. We have attempted to clarify as follows: (line 185–198)

Fibrosis distributions in the volumetric geometries were interpolated from LGE maps, We used a priority-based protocol ensuring that the total fibrotic burdens matched those reported by Merisight. [...] Effectively, this amounted to the identification of a lower cutoff value for normalized LGE in order for a element to be included. In practice, this generally resulted in expansion of fibrotic areas, although this was not necessarily uniform since the approach was based on normalized intensity as opposed to geometric region growing.

- Is a different fiber architecture expected in fibrotic areas? If so, does the rule-based fiber direction method account for this?

  We did not alter the fiber architecture in fibrotic areas. We have clarified this as following (line 209–210):

  The same fiber architecture was used in both baseline and fibrotic simulations.

  However, changes in anisotropy (both ways) were imposed through independently changing conduction value parameters $\mathrm{CV_L}$ and $\mathrm{CV_T}$ on the electrophysiological side, and stiffness parameters a and a_f on the mechanical side. None of these appeared to have prominent impact in the metrics considered.

- "Active tension generation was scaled with 50 kPa at baseline, calibrated to give approximately 30% emptying fraction". Was Cine MRI obtained? If so, did these data show a similar emptying fraction, and are any differences between patients observed/expected? It would be helpful to show the data in Fig. 3 (middle row), either full cycle or end-diastolic volume to indicate how well the model was calibrated for each individual patient.

  Cine MRI was indeed obtained, however, we only have total EF (active and passive combined, and not over time). Extraction of minimum and maximum volumes from the clinical images was not feasible in our system. Nevertheless, we agree that these are key metrics and we now also report emptying fraction in Table 1 and simulation-predicted EF values with baseline model parameterization as follows (line 438–442):

  Based on volumetric measurements at different stages, we also computed model-predicted LAEF for comparison with clinical values (reported in Table 1). Total LAEFs at baseline were 33.70% for Patient 1, 38.13% for Patient 2, and 37.52%; consistently lower than corresponding clinical estimates. Simulation-predicted active LAEFs were 23.99%, 29.37%, and 33.97%, while passive LAEFs were 12.78%, 12.16%, and 16.61% for Patients 1, 2, and 3.

  With our current LA model implementation, we can relatively easily scale the active part, but not the passive part (while keeping values within physiological ranges). We are working to resolve this limitation for future studies, as noted in the revised discussion (line 762–765):

  LAEF values in our simulations (33.70%, 38.13%, and 44.94% at baseline) were consistently lower than corresponding clinical estimates (39.24%, 51.56%, 58.2%; see Table 1). The discrepancy can be attributed to the passive part of the LAEF, which was underestimated in our model (12.78%, 12.16%, 16.61% vs. reference values of 35–40% [7, 8]), whereas active LAEFs agreed with typical normal values (23.99%, 29.57%, 33.97% vs. 30%). In the future, model improvements can be made to better represent passive function. Such improvements might also increase the sensitivity of the conduit function, which was generally found to be low across all tracked metrics in our experiments.

- FFD is an elegant approach to efficiently investigate parameter sensitivity. Recent work (for example: Strocchi et al., Longobardi et al., Jones & Oomen) leveraged surrogate models to perform a global sensitivity analysis on (electro-)mechanical cardiac model. It would be helpful to comment on how FFD differs from global methods like Sobol's method beyond computational cost.

  We agree with the reviewer that there are more variables to consider than computational efficiency alone. FFD and global approaches like Sobol's method differ in ways that make scope and cost

difficult to disentangle: global methods are more expensive precisely because they provide more comprehensive sensitivity information. Surrogate modeling methods like Gaussian process emulation can help reduce this cost but their effectiveness depends on how well the surrogate captures complex, nonlinear system behavior. We have tried to expand on these aspects. The revised paragraphs, with corrections highlighted (and new citations added), now reads (line 664–680):

Sensitivity analysis can be performed in various ways, involving many methodological choices. Advanced techniques like the Morris elementary effects method [9, 10, 11], Sobol indices [12, 13, 14, 15], and Bayesian history matching [16, 17] involve finer interval sampling, capturing non-linear behavior, interaction effects, and sensitivity regions. However, these benefits come at the cost of running more simulations. These can feasibly be carried out through model simplifications, computational optimization strategies, or surrogate models like Gaussian process emulators [18, 13, 14, 16, 17].

In our study, we sampled all relevant parameters at two levels: baseline and fibrotic. The FFD scheme presented an efficient methodology allowing us to perform our sensitivity analysis while accounting for interaction with a detailed multi-scale, multi-physics computational model, based on a manageable number of simulations. It enables direct quantification and assessment of the directionality of parameter effects, which is not provided directly through Sobol analysis nor Bayesian history matching, and only statistically approximated in the Morris elementary effects method. Finally, while OFAT is a rudimentary method that (by definition) misses all interactive effects, this attribute makes it an eminently interpretable approach.

- Could the reduced-order approach for the other chambers have contributed to the differences in findings compared to Strocchi et al., who used a four-chamber cardiac model?

  We would like to thank the reviewer for the suggestion. Differences might indeed arise due to limitations of the simplified representation of the other chambers, along with other differences in model representation. We have expanded on this limitation as follows (line 741–748):

  Similarly, the use of a lumped 0D model representing the impact of the other chambers, rather than a full 4-chamber model, might not accurately capture interaction between the different chambers [19]. Including at least the left ventricle could better represent the passive phase of atrial function and ventricular remodeling. However, this would greatly increase computational costs and model complexity. A potential future compromise would be to estimate patient-specific values for the CircAdapt parameters corresponding volumes of each cardiac chamber, whereas our present study used default settings for the sake of simplicity.

- While I appreciate the study is not statistically powered to identify potential sex differences, are any sex differences expected in atrial fibrotic remodeling?

  We agree with the reviewer that this is an important point to keep in mind, and it might guide future research project questions. We have added the following to the discussion to address this aspect (line 699–703):

  [...] Among AF patients, males have larger atria (but not higher LA volume indices), whereas females have higher fibrosis burden [20] and larger low-voltage areas [21]. This is the opposite of what we observe for Patient 1 (female, largest LA) and Patient 2 (male, highest fibrosis burden), but not unexpected given inter-individual variability.

  Minor comments:

- There is a broken link on line 296.

  We have fixed the broken link (now line 331):

  "[...] are listed in S1 Appendix (§ A.2, Table A1)."

- What causes the oscillations in the bottom right of the PV loops?

  The oscillations happen when we have something close to a pressure equilibrium between the LV and the LA, causing fluctuation. We have commented on this as follows (line 427–431):

  The A-loop was larger than the P-loop in area, indicating higher changes in volume and pressure. Prior to atrial contraction (when we were close to an equilibrium between atrial and ventricular pressure), we observed small oscillations in both volume and pressure, also are visible in the lower part of the V-loop.

- Fig. 9b: legend's red line is mislabeled.

  Thanks to the reviewer for catching that; we have corrected the legend.

Reviewer 2

The manuscript by Telle et al. presents an in silico investigation of atrial electromechanical abnormalities following fibrotic remodeling based on person-specific patterns. Nine parameters were investigated alone or in combination to understand their contribution to different metrics of atrial performance. This is an important problem as number of AF patients increases.

- The most striking result was that reduction of IK1 improves atrial performance (increases A-loop and dampens the adverse effects of reduced ICa2+). This result is somewhat paradoxical and requires more in depth discussion. Please elaborate on the mechanism by which IK1 enlarges the A-loop. Reducing IK1 depolarizes the resting membrane potential, which may be pro-arrhythmic by itself, or lead to undesired effects. Blockers of IK1 can indeed reduce excitability and be anti-arrhythmic, but dosing these to the right concentration to not trigger ectopic rhythm is tricky. This finding has to be put into context.

  We agree with the reviewer that this is a striking and somewhat unexpected result. We have now expanded the first sections in the Discussion as follows (line 581–585):

  [...] In simulations with impaired I_{K1}, we observed an elevated membrane potential and an increase in intracellular calcium transient amplitude (Fig 8), leading to enhanced active tension and improved LA function. This effect mitigated the impairing impact of fibrosis from other factors.

  We also expanded on the arrhythmogenic perspective of this in the the Future work section, as follows (line 798–804):

  [...] However, blocking I_{K1} may trigger ectopic activity due to increased resting membrane potential. Careful investigation is warranted regarding the balance between arrhythmic effects and benefit to atrial biomechanical function. An interesting avenue and extension of our work could be exploring the impact of rhythm control medication on atrial and ventricular function, in both sinus rhythm and subject to re-entry waves mimicking those observed in atrial and ventricular fibrillation.

- Perhaps another somewhat surprising finding is the very modest effect of stiffness on the metrics for atrial performance in this study. Please comment in more detail how these predictions compare to others and the clinical observations and what difference in assumptions exist between computational studies (some of that is covered in the Discussion yet can be clarified further). What are the limitations of the currently used lumped parameter model of hemodynamics (0D model)?

  We agree with the reviewer, this is an interesting point. We believe the limited impact in our study can be explained by the fact that we only increased stiffness in fibrotic regions, while other studies have imposed the impact globally. The use of the lumped parameter model may indeed come into play in this aspect of our findings, especially with regards to the passive function, but we still believe that tissue heterogeneity is the main explanation for the modest impact of stiffness.

We have expanded on these perspectives as follows (line 619–653):

Stiffness has also been explored in other models. Moyer et al. [22] found that fibrosis-associated increase in global stiffness reduced both systolic and diastolic function, with increased pressure and reduced volume for the latter. Meskin et al. [23] reported that higher compliance (lower stiffness) decreased pressure and increased A-loop area. Both studies advanced understanding of LA biomechanics but applied uniform stiffness changes, ignoring heterogeneity introduced by fibrotic remodeling. They also differed in material representation: our model uses a reduced Holzapfel-Ogden law with a conditional term (combined with detailed fiber orientations), while the formulation used by Moyer et al. increased fiber-direction stiffness under both contraction and stretch, and Meskin et al. used silicone rubber, which is isotropic. Pericardial boundary conditions in our model may further dampen stiffness effects. We found that increased stiffness in fibrotic regions minimally reduced A-loop area, likely because changes were confined to fibrotic regions, while slightly lowering diastolic pressure, probably due to altered mitral valve flow. Gonzalo et al. [24] observed a larger systolic volume reduction (5% vs. 2%), likely reflecting the fivefold stiffness increase in fibrotic regions, while diastolic volume remained unchanged. Physiologically, non-fibrotic regions are also probably stiffened due to interstitial fibrosis, though substantial stiffness heterogeneity remains. It seems plausible that changes in stiffness governing the largest part of the atrial body – perhaps better assessed via a median rather than mean value – will govern the relative impact on LA function.

A shift in LA volume combined with increased stiffness has been observed in clinical studies [25], although this likely included volumetric expansion. LA stiffness indices derived from clinical flow and strain measurements show predictive power [26, 25, 27], but not independently of volumetric indices. Lamy et al. [28] used invasive measurements of pressure to calculate the stiffness index and correlated it with fibrotic burden assessed by LGE. They found significant differences in LA strain metrics, but notably not in pressure. Further clinical, experimental, and computational studies are needed to better understand the relationships between LA stiffness, volume, and pressure changes.

Furthermore, we have commented on our use of a lumped parameter models under limitations (line 741–748):

Similarly, the use of a lumped 0D model representing the impact of the other chambers, rather than a full 4-chamber model, might not accurately capture interaction between the different chambers [19]. Including at least the left ventricle could better represent the passive phase of atrial function and ventricular remodeling. However, this would greatly increase computational costs and model complexity. A potential future compromise would be to estimate patient-specific values for the CircAdapt parameters corresponding volumes of each cardiac chamber, whereas our present study used default settings for the sake of simplicity.

Other questions and suggestions:

- In the Abstract, would be better to either avoid or define terms (e.g. A-loop) that are not commonly used outside a specific area, for clarity to a broader audience.

We see the reviewer's point here. We could do either, but opted for defining A-loop area, and also moved the sentence about spatiotemporal analysis up so it comes in the same order as the results section. The abstract has been updated as follows (line 31–45):

Impairment of L-type calcium current (I_{CaL}) was most detrimental (up to 64% reduction in A-loop area of the left atrial pressure-volume relationship, quantifying work performed during atrial contraction). Conversely, reduced inward rectifier current (I_{K1}) led to improved atrial function (up to 27% increase in A-loop area). Detailed analysis of spatiotemporal distributions linked these effects to changes in intracellular calcium handling. [...]

- Are the listed conduction velocities correct and are they average values, incl. fibrotic regions? They seem low, unless the units are different.

  We are very thankful to the reviewer for catching this error on our part. First off, the units should be m/s, not cm/s. This change brings the values into a much more plausible range. Furthermore, we checked our simulations to confirm correctness and found another inconsistency: For Patient 3 we have corrected CV_L from 0.8100 m/s to 0.835 m/s and for CV_T from 0.3614 m/s to 0.373 m/s (the old values were earlier estimations for the same patient).

  With that corrected, these are indeed average values, which we now have specified as follows (line 227–228):

  Reported values were representative for the whole tissue, without any adjustments for fibrotic regions.

  We acknowledge that it could have made more sense to do the parameterization with the fully fibrotic setup, however (as stated in the discussion under limitations) we don't believe this simplification make any noticeable difference.

- Was the fiber direction personalized per patient or generic? Would you expect that to play a role for better prediction?

  The fiber direction was generic. We have specified this as following: (Line 207–210)

  Fiber direction maps (delineating the longitudinal myocyte directions) were generated for each geometry using a rule-based method defined by LA landmarks, similar to those described in previous publications [29, 30]. The same fiber architecture was used in both baseline and fibrotic simulations.

  and mentioned personalized fiber direction as a possibility for future studies in the Discussion (line 685–688):

  Additionally, myofiber architecture was specified using a rule-based algorithm. Cardiac computed tomography offers the ability to quantify regional wall thickness and patient-specific myofiber orientation [6]. Incorporating such data could enhance model accuracy and predictive value.

  While personalized fiber directions would be preferred compared to the generic, and we would have used those if we had them, we don't anticipate a particular high sensitivity in fiber direction compared to other generic parameters (e.g., wall thickness, myocardial stiffness, pressure).

- In Figure 9B, the legend needs to be corrected for the red line to IK1.

  Thanks to the reviewer for catching that; we have corrected the legend.

Other changes

We have also carefully revised the document for any inconsistencies in reported values and wording, aiming for correctness and consiseness. These have also been marked in the main document. Major updates include:

- Volumetric measurements: In the process of revising the paper, we noticed an inconsistency in the way we originally calculated clinical estimates of LA end-diastolic volume. Relevant values are now updated and are correct (see Table 1). We removed corresponding annotations in Figure 1 to improve figure readability. Additionally, we expanded on the slight disparity between clinically estimated and model-predicted volumes as follows (line 293–299):

  The simulated end-diastolic volumes were slightly higher than the corresponding clinical measurements (Table 1; Fig 3, middle row). This was expected given that the volume computations in the simulations were performed on a closed atrial cavity that included the pulmonary vein remnants,

see Fig 1. Specifically, the simulations yielded end-diastolic LA volumes of 124.2 mL, 130.9 mL, and 63.4 mL, compared to clinically measured volumes of 110.0 mL, 112.5 mL, and 60.0 mL. This systematic offset was consistent with the modeling approach and demonstrated an overall agreement between simulated and clinical data.

- Scaling factors for I_{CaL} and I_{Na} were corrected twice (their values had been swapped).
- We added units for the CV table (i.e., "m/s") in the Appendix (not highlighted).

References

[1] Nadine Kawel-Boehm, Scott J Hetzel, Bharath Ambale-Venkatesh, Gabriella Captur, Christopher J Francois, Michael Jerosch-Herold, Michael Salerno, Shawn D Teague, Emanuela Valsangiacomo-Buechel, Rob J Van der Geest, et al. Reference ranges ("normal values") for cardiovascular magnetic resonance (cmr) in adults and children: 2020 update. *Journal of Cardiovascular Magnetic Resonance*, 22(1):87, 2020.

[2] Pedro Silva Cunha, Sérgio Laranjo, Sofia Monteiro, Inês Grácio Almeida, Tiago Mendonça, Iládia Fontes, Rui Cruz Ferreira, Ana G Almeida, Maxim Didenko, and Mário Martins Oliveira. Left atrial wall thickness estimated by cardiac ct: Implications for catheter ablation of atrial fibrillation. *Journal of Clinical Medicine*, 13(18):5379, 2024.

[3] Flemming Javier Olsen, Litten Bertelsen, Niels Vejlstrup, Søren Zöga Diederichsen, Caroline Løkke Bjerregaard, Claus Graff, Axel Brandes, Derk Krieger, Ketil Haugan, Lars Køber, et al. Association between four-dimensional echocardiographic left atrial measures and left atrial fibrosis assessed by left atrial late gadolinium enhancement. *European Heart Journal-Cardiovascular Imaging*, 24(1):152–161, 2023.

[4] Longchang Li, Lijun Li, Dezhi Yang, Shuxiong Nong, Cheng Luo, and Chun Gui. Analysis of the thickness characteristics of the left atrial posterior wall and its correlation with the low and no voltage areas of the left atrial posterior wall in patients with atrial fibrillation. *Journal of Cardiothoracic Surgery*, 19(1):187, 2024.

[5] Wei Liu, Shijie Li, Lina Dou, Chunai Hu, and Bing Han. Identification of left atrial wall thickness in substrate mapping of atrial fibrillation. *Frontiers in Cardiovascular Medicine*, 12:1592532, 2025.

[6] Neil Bodagh, Michelle C Williams, Keeran Vickneson, Ali Gharaviri, Steven Niederer, and Steven E Williams. State of the art paper: Cardiac computed tomography of the left atrium in atrial fibrillation. *Journal of cardiovascular computed tomography*, 17(3):166–176, 2023.

[7] Alicia M Maceira, Juan Cosin-Sales, Sanjay K Prasad, and Dudley J Pennell. Characterization of left and right atrial function in healthy volunteers by cardiovascular magnetic resonance. *Journal of Cardiovascular Magnetic Resonance*, 18(1):64, 2016.

[8] Vien T Truong, Cassady Palmer, Sarah Wolking, Brandy Sheets, Michael Young, Tam NM Ngo, Michael Taylor, Sherif F Nagueh, Karolina M Zareba, Subha Raman, et al. Normal left atrial strain and strain rate using cardiac magnetic resonance feature tracking in healthy volunteers. *European Heart Journal-Cardiovascular Imaging*, 21(4):446–453, 2020.

[9] Daniel E Hurtado, Sebastián Castro, and Pedro Madrid. Uncertainty quantification of 2 models of cardiac electromechanics. *International Journal for Numerical Methods in Biomedical Engineering*, 33(12):e2894, 2017.

[10] Virginie Le Rolle, Elena Galli, David Danan, Karim El Houari, Arnaud Hubert, Erwan Donal, and Alfredo I Hernández. Sensitivity analysis of a left ventricle model in the context of intraventricular dyssynchrony. *Acta Biotheoretica*, 68(1):45–59, 2020.

[11] Marion Taconné, Kimi P Owashi, Elena Galli, Jürgen Duchenne, Arnaud Hubert, Erwan Donal, Alfredo I Hernàndez, and Virginie Le Rolle. Model-based analysis of myocardial strains in left bundle branch block. *Frontiers in Applied Mathematics and Statistics*, 8:833003, 2022.

[12] Pras Pathmanathan, Jonathan M Cordeiro, and Richard A Gray. Comprehensive uncertainty quantification and sensitivity analysis for cardiac action potential models. *Frontiers in physiology*, 10:721, 2019.

[13] Elias Karabelas, Stefano Longobardi, Jana Fuchsberger, Orod Razeghi, Cristobal Rodero, Marina Strocchi, Ronak Rajani, Gundolf Haase, Gernot Plank, and Steven Niederer. Global sensitivity analysis of four chamber heart hemodynamics using surrogate models. *IEEE Transactions on Biomedical Engineering*, 69(10):3216–3223, 2022.

[14] Alan Lazarus, David Dalton, Dirk Husmeier, and Hao Gao. Sensitivity analysis and inverse uncertainty quantification for the left ventricular passive mechanics. *Biomechanics and Modeling in Mechanobiology*, 21(3):953–982, 2022.

[15] Marina Strocchi, Stefano Longobardi, Christoph M Augustin, Matthias AF Gsell, Argyrios Petras, Christopher A Rinaldi, Edward J Vigmond, Gernot Plank, Chris J Oates, Richard D Wilkinson, et al. Cell to whole organ global sensitivity analysis on a four-chamber heart electromechanics model using gaussian processes emulators. *PLoS Computational Biology*, 19(6):e1011257, 2023.

[16] Stefano Longobardi, Alexandre Lewalle, Sam Coveney, Ivar Sjaastad, Emil KS Espe, William E Louch, Cynthia J Musante, Anna Sher, and Steven A Niederer. Predicting left ventricular contractile function via gaussian process emulation in aortic-banded rats. *Philosophical Transactions of the Royal Society A*, 378(2173):20190334, 2020.

[17] Clara E Jones and Pim JA Oomen. Synergistic biophysics and machine learning modeling to rapidly predict cardiac growth probability. *Computers in Biology and Medicine*, 184:109323, 2025.

[18] Marina Strocchi, Christoph M. Augustin, Matthias A. F. Gsell, Elias Karabelas, Aurel Neic, Karli Gillette, Caroline H. Roney, Orod Razeghi, Jonathan M. Behar, Christopher A. Rinaldi, Edward J. Vigmond, Martin J. Bishop, Gernot Plank, and Steven A. Niederer. The Effect of Ventricular Myofibre Orientation on Atrial Dynamics. In *Functional Imaging and Modeling of the Heart: 11th International Conference, FIMH 2021, Stanford, CA, USA, June 21-25, 2021, Proceedings*, page 659–670, Berlin, Heidelberg, 2021. Springer-Verlag.

[19] Aaron L Brown, Ju Liu, Daniel B Ennis, and Alison L Marsden. Cardiac mechanics modeling: recent developments and current challenges. *arXiv preprint arXiv:2509.07971*, 2025.

[20] Nazem Akoum, Christian Mahnkopf, Eugene G Kholmovski, Johannes Brachmann, and Nassir F Marrouche. Age and sex differences in atrial fibrosis among patients with atrial fibrillation. *EP Europace*, 20(7):1086–1092, 2018.

[21] Olivier Van Leuven, Marco Bergonti, Francesco Raffaele Spera, Teba Gonzalez Ferrero, Michelle Nsahlai, Giada Bilotta, Maxime Tijskens, Wim Boris, Johan Saenen, Wim Huybrechts, et al. Gender-related differences in atrial substrate in patients with atrial fibrillation. *The American Journal of Cardiology*, 203:451–458, 2023.

[22] Christian B Moyer, Patrick T Norton, John D Ferguson, and Jeffrey W Holmes. Changes in global and regional mechanics due to atrial fibrillation: insights from a coupled finite-element and circulation model. *Annals of Biomedical Engineering*, 43:1600–1613, 2015.

[23] Masoud Meskin, Philip Alexander Starkey, Alexander Emil Kaspersen, Steffen Ringgaard, Signe Gram Sand, Jens Vinge Nygaard, Jørgen Arendt Jensen, Marie Sand Traberg, and Peter Johansen. Investigating the importance of left atrial compliance on fluid dynamics in a novel mock circulatory loop. *Scientific Reports*, 14(1):1864, 2024.

[24] Alejandro Gonzalo, Christoph M Augustin, Savannah F Bifulco, Åshild Telle, Yaacoub Chahine, Ahmad Kassar, Manuel Guerrero-Hurtado, Eduardo Durán, Pablo Martínez-Legazpi, Oscar Flores, et al. Multiphysics simulations reveal haemodynamic impacts of patient-derived fibrosis-related changes in left atrial tissue mechanics. *The Journal of Physiology*, 602(24):6789–6812, 2024.

[25] Irfan M Khurram, Farhan Maqbool, Ronald D Berger, Joseph E Marine, David D Spragg, Hiroshi Ashikaga, Vadim Zipunnikov, David A Kass, Hugh Calkins, Saman Nazarian, et al. Association between left atrial stiffness index and atrial fibrillation recurrence in patients undergoing left atrial ablation. *Circulation: Arrhythmia and Electrophysiology*, 9(3):e003163, 2016.

[26] Hideyuki Kishima, Takanao Mine, Eiji Fukuhara, Kenki Ashida, and Masaharu Ishihara. The association between left atrial stiffness and low-voltage areas of left atrium in patients with atrial fibrillation. *Heart and Vessels*, 34(11):1830–1838, 2019.

[27] Darae Kim, Jeong Hun Seo, Ki Hong Choi, Seung Hun Lee, Jin-Oh Choi, Eun-Seok Jeon, and Jeong Hoon Yang. Prognostic implications of left atrial stiffness index in heart failure patients with preserved ejection fraction. *Cardiovascular Imaging*, 16(4):435–445, 2023.

[28] Jérôme Lamy, Roy Taoutel, Romy Chamoun, Joseph Akar, Steven Niederer, Hamid Mojibian, Steffen Huber, Lauren A Baldassarre, Judith Meadows, Dana C Peters, et al. Atrial fibrosis by cardiac mri is a correlate for atrial stiffness in patients with atrial fibrillation. *The International Journal of Cardiovascular Imaging*, 40(1):107–117, 2024.

[29] Luca Azzolin, Martin Eichenlaub, Claudia Nagel, Deborah Nairn, Jorge Sánchez, Laura Unger, Thomas Arentz, Dirk Westermann, Olaf Dössel, Amir Jadidi, et al. Augmenta: Patient-specific augmented atrial model generation tool. *Computerized Medical Imaging and Graphics*, 108:102265, 2023.

[30] Elena Zappon, Luca Azzolin, Matthias AF Gsell, Franz Thaler, Anton J Prassl, Robert Arnold, Karli Gillette, Mohammadreza Kariman, Martin Manninger-Wünscher, Daniel Scherr, et al. An efficient end-to-end computational framework for the generation of ecg calibrated volumetric models of human atrial electrophysiology. *arXiv preprint arXiv:2502.03322 [Preprint]*, 2025.
